# Informed community mobilization for dengue prevention in households with and without a regular water supply: Secondary analysis from the Camino Verde trial in Nicaragua

**DOI:** 10.1186/s12889-017-4295-7

**Published:** 2017-05-30

**Authors:** Alvaro Cárcamo, Jorge Arosteguí, Josefina Coloma, Eva Harris, Robert J. Ledogar, Neil Andersson

**Affiliations:** 1 CIET Nicaragua, Managua, Nicaragua; 2 Division of Infectious Diseases and Vaccinology, School of Public Health, University of California, Berkeley, Berkeley, CA USA; 3 CIET International, New York, NY USA; 4 Centro de Investigación de Enfermedades Tropicales, Universidad Autónoma de Guerrero, Acapulco, Mexico; 5 Department of Family Medicine, McGill University, Montreal, Canada

**Keywords:** Water supply, *Aedes aegypti*, Intermittent water supply

## Abstract

**Background:**

Studies in different countries have identified irregular water supply as a risk factor for dengue virus transmission. In 2013, *Camino Verde,* a cluster-randomised controlled trial in Managua, Nicaragua, and Mexico’s Guerrero State, demonstrated impact of evidence-based community mobilisation on recent dengue infection and entomological indexes of infestation by *Aedes aegypti* mosquitoes. This secondary analysis of data from the trial impact survey asks: (1) what is the importance of regular water supply in neighbourhoods with and without the trial intervention and (2) can community interventions like *Camino Verde* reasonably exclude households with adequate water supply?

**Methods:**

Entomological data collected in the dry season of 2013 in intervention and control communities allow contrasts between households with regular and irregular water supplies. Indicators of entomological risk included the House Index and pupa positive household index. Generalised linear mixed models with cluster as a random effect compared households with and without regular water, and households in intervention and control communities.

**Results:**

For the House Index, regular water supply was associated with a protection in both intervention households (OR 0.7, 95%CI 0.6–0.9) and control households (OR 0.6, 95%CI 0.5–0.8). For the pupa positive household index, we found a similar protection from regular water supply in intervention households (OR 0.6, 95%CI 0.4–0.8) and control households (OR 0.7, 95%CI 0.5–0.9). The *Camino Verde* intervention had a similar impact on House Index in households with regular water supply (OR 0.7, 95%CI 0.5–1.0) and irregular water supply (OR 0.6, 95%CI 0.4–0.8); for the pupa positive household index, the effect of the intervention was very similar in households with regular (OR0.5, 95%CI 0.3–0.8) and irregular (OR 0.5, 95%CI 0.3–0.9) water supply.

**Conclusion:**

While *Aedes aegypti* control efforts based on informed community mobilisation had a strong impact on households without a regular water supply, this intervention also impacted entomological indices in households with a regular water supply. These households should not be excluded from community mobilisation efforts to reduce the *Aedes aegypti* vector.

**Trial registration:**

ISRCTN27581154.

## Background

Water is a necessary condition for health, but also a route for disease transmission [1], mainly when there are supply problems. Many urban centres lack adequate water supply, with services available only for a limited time or in restricted areas of the city. This makes household water storage necessary to meet needs when the service is unavailable [2]. In Managua, household water is stored mainly in barrels, but also in buckets and jugs. The stored water is used for personal hygiene, cooking and household cleaning chores. One risk of these water stores is that improperly handled containers become favourable environments for the eggs, larvae and pupae of the *Aedes aegypti* mosquito, resulting in an increase of new adult mosquito populations.

Several studies have explored the links between water supply, *Aedes aegypti* ecology and dengue transmission risk. A 1992 study in Venezuela’s coastal region showed interruptions of the water supply were associated with more *Aedes aegypti* larvae and pupae in water storage containers [3]. Another study in Maracay, Venezuela, showed adequate water supply was a protective factor against dengue [4]. A 2005 anthropological study in Fortaleza, Brazil, pointed to water supply affecting *Aedes aegypti* ecology [5]. A 2010 cohort study of 75,000 households in Vietnam linked frequency of water supply and population density with dengue transmission risk [6].

Managua, Nicaragua’s capital and main commercial and service centre, is less than 100 m above sea level. Its tropical climate reaches 38° Celsius during the warmest seasons [7]. The National Water and Sewerage Company (ENACAL, *Empresa Nacional de Acueductos y Alcantarillados*) runs and manages the system distributing water to 1.5 million residents [8]. According to ENACAL data, the water distribution network reached a nominal 94% of Managua’s population in 2012 [9]. However, hourly coverage deficiencies result from planned service outages and technical or operational problems in the network. Understanding of the system’s effective coverage requires information from householders themselves. The water supply is affected by topography, so may be regular for some households and irregular for others within the same neighbourhood.

Between 2010 and 2013, the *Camino Verde* (Green Way) randomised controlled trial in Managua and Guerrero State, Mexico, reported a significant impact of informed community mobilization on *Aedes aegypti* larvae and pupae counts [10]. We present here a secondary analysis of data from the impact survey of the trial in Managua in the 2013 dry season, addressing two questions: 1) what is the importance of regular water supply in neighbourhoods with and without the trial intervention and 2) can community interventions like *Camino Verde* safely exclude households with adequate water supply?

## Methods

In the trial impact survey in the 2013 dry season, field teams visited households in 60 neighbourhoods (30 intervention and 30 control). This article uses data obtained from all 60 neighbourhoods during this survey.

### Entomology inspections

Six field teams of 12 workers each carried out the household survey, which included entomological inspections. Each team included five entomological reviewers and two supervisors, one of whom either had responsibilities in the vector control programme of the local healthcare system (known by its Spanish acronym as the SILAIS) or was an entomologist in the Ministry of Health’s National Diagnostics and Reference Centre (CNDR). The entomological review applied the protocol and techniques standardised by the vector control programme for collecting, transporting, conserving, identifying, counting and classifying immature *Ae. aegypti* specimens at different stages of development. Each reviewer inspected every water container in each household to detect and to capture larvae and pupae. They classified containers as barrels or large tubs, buckets, washtubs, plant pots, flower pots, tyres, non-storage containers (pails, drinking fountains, etc.), and discarded household items that could hold water (*calaches*).

### Handling of specimens

The inspectors placed all larvae or pupae in labelled jars containing 70% alcohol; they transferred the jars to the CNDR entomology laboratory, where a team of specialised entomologists verified and classified the specimens. Receptacles were classified positive when CNDR evaluators found one or more immature forms of mosquito at any stage of development. The entomology team also counted the collected specimens and classified them by stage of development, as an indicator of each container’s mosquito productivity.

### Outcomes

We calculated two entomological indices, one to reflect all immature forms and one to reflect just pupae. The House Index (HI) is the proportion of households positive for larvae or pupae. We classified a household as positive for pupae if the entomology lab confirmed the presence of one or more pupae in that household. Because the exposure of interest was household water supply (see below), the Breteau, container and pupae per person indices could not be applied in their original form.

### Exposure

Our measure of regularity of water supply came from answers to the question "How many times did the household lack water in the last week?” We classified water supply as “regular” if a household had continuous access to water or lacked it for no more than a day during the week prior to the survey. We classified households reporting no water supply for two or more days in the week prior to the survey as having “irregular” water supply.

### Statistical analysis

The *Camino Verde* trial used cluster as the unit of principal analysis [10]. This secondary analysis used household as the unit because regularity of water supply was not uniform within the clusters. Bivariate and then multivariate analysis evaluated impact of regularity of water supply on each entomological index, in the context of other factors that might affect the outcome, derived from household responses to an administered questionnaire. Sensitivity analysis for the outcome of pupae positive households converted the continuous variable of regularity of water supply into a binomial variable by categorizing at two additional points, and rerunning the models.

Bivariate analysis examined the association between the two entomological indicators as outcomes, and exposure to regular water supply, as well as other variables potentially related to the outcomes, derived from household responses. We included variables significant at the 5% level in bivariate analysis (essentially temephos and water supply) in a saturated generalised linear mixed model (GLMM) with cluster as a random effect. We developed separate models for intervention and control sites to assess the importance of water supply in relation to informed community mobilisation. To address the overall importance of the intervention, we developed separate models for regular and irregular water supply, with cluster as a random effect. We report the odds ratio (OR) and protection (1-OR) from these analyses, along with the number needed to treat (NNT). The analysis used the Zelig programme in R through CIETmap, an open-source package that provides an interface with the R statistical programming language [11]. We used the Zelig default burn-in, discarding the first 1000 iterations.

## Results

Among all households for which we had water supply data in January 2013 (*n* = 8121), 50% had continuous access to water or lacked it for no more than a day during the week prior to the survey (regular water, 4075/8121) and the other half reported interruption of water supply for two or more days in the week prior to the survey (“irregular” water, 4046/8121). Data on water supply were missing from 15 households, which were excluded from the analysis. The rates of regular supply were similar in intervention (2041/4046) and control (2034/4075) neighbourhoods.

### Effect of water supply in intervention and control households

Households with an irregular water supply were roughly twice as likely to have a positive House Index and to be pupae-positive compared with households with a regular supply (Table 1). Having regular water was associated with a protection (1-OR) of 30% for the House Index and 40% for the pupae-positive household index. For both entomological outcomes, the effect of regularity of water supply was similar in intervention and control households (Tables 2 and 3). Contrasting with the similar odds ratio in intervention and control households, the number needed to treat (with a regular water supply) was quite different in intervention and control neighbourhoods: to prevent one household having a positive Household Index (any larvae or pupae) in each neighbourhood, 29 intervention households or 14 control households would need to be provided with a regular water supply. To convert one household from pupae-positive to pupae-negative in intervention and control neighbourhoods, 46 intervention households or 33 control households would need to be provided with a regular water supply.

**Table 1:** Entomological indices and regularity of water supply, Managua, January 2013

Water supply	No. households	House Index^a^		Pupae	
		Positive households	House Index (%)	N	Mean per household
Regular	4075	565	14	1314	0.3
Irregular	4046	968	24	3496	0.9

^a^House Index = percent of houses infested with larvae and/or pupae

**Table 2:** House Index in households with regular and irregular water supply, in intervention and control sites, Managua, January 2013

Water supply	Intervention sites		Control sites		Impact of intervention OR (95% CI of OR); NNT (95% CI of NNT)^a^
	No. households	No. (%) positive for larvae/pupae	No. households	No. (%) positive for larvae/pupae	
Regular supply	2028	243 (12)	2031	396 (20)	0.7 (0.5–1.0); 24 (12–251)
Irregular supply	2015	322 (16)	2016	572 (28)	0.6 (0.4–0.8); 11 (7–36)
Association with regularity of water supply: OR (95% CI of OR); NNT (95% CI of NNT)^a^					
All sites	Intervention sites		Control sites		
0.7 (0.6–0.8); 19 (13–35)	0.7 (0.6–0.9); 29 (16–103)		0.6 (0.5–0.8); 14 (9–24)		

^a^Odds Ratio and 95% confidence interval; number needed to treat and 95% confidence interval. Both OR and NNT from GLMM, with cluster as random effect. In addition to regularity of water supply or intervention status, observed presence of temephos remained in the final models

**Table 3:** Pupa positive households with regular and irregular water supply, in intervention and control sites, Managua, January 2013

Water supply	Intervention sites		Control sites		Impact of intervention* OR (95% CI of OR); NNT (95% CI of NNT)
	No. households	No. (%) positive for pupae	No. households	No. (%) positive for pupae	
Regular supply	2028	76 (4)	2031	135 (7)	0.5 (0.3–0.8); 30 (18–83)
Irregular supply	2015	148 (7)	2016	248 (12)	0.5 (0.3–0.9); 22 (12–77)
Association with regularity of water supply: OR (95% CI of OR); NNT (95% CI of NNT)					
All sites	Intervention sites		Control sites		
0.6 (0.5–0.8); 40 (28–84)	0.6 (0.4–0.8); 46 (26–134)		0.7 (0.5–0.9); 32 (19–85)		

*Odds Ratio and 95% confidence interval; number needed to treat and 95% confidence interval. Both OR and NNT from GLMM, with cluster as random effect. In addition to regularity of water supply or intervention status, observed presence of temephos remained in the final models

### Informed community mobilisation in households with regular and irregular water supply

We examined the impact of the informed community mobilisation intervention on *Aedes aegypti* larvae and pupae in separate models by water supply, again with cluster as a random effect in the GLMM (Tables 2 and 3). The *Camino Verde* intervention had a similar protective impact on Household Index in households with regular (OR 0.7, 95%CI 0.5–0.1) and irregular (OR 0.6, 95%CI 0.4–0.8) water supply; for the pupa positive household index, the effect of the intervention was very similar in households with regular (OR0.5, 95%CI 0.3–0.8) and irregular (OR 0.5, 95%CI 0.3–0.9) water supply. For both entomological outcomes, however, the NNT was lower in households with irregular water supply (Tables 2 and 3): the *Camino Verde* intervention would need to reach 11 households (95% CI 7–36) with irregular water supply or 24 households (95% CI 12–251) with regular water supply to prevent one household having a positive House Index; to reduce the pupa index in one house, the intervention would need to include 30 houses with regular water and only 22 with irregular water.

A sensitivity analysis looked at different cut-off points of water supply regularity. Moving the cut-off one place to the right (less stringent) in a range of 1–5 produced an odds ratio of 0.6 and First Difference of 0.026; moving it one place to the left (more stringent) produced an odds ratio of 0.6 and First Difference of 0.025. The result of the cut-off used in the analysis was an odds ratio of 0.6 and First Difference of 0.028, implying the cut-off point does not account for a spurious result.

## Discussion

Confirming findings of other studies, our results indicate a roughly twofold increased risk for entomological evidence of *Aedes aegypti* infestation in households with irregular water supply compared with those that have regular water supply. Water supply affected entomological risk in both intervention and control clusters in the *Camino Verde* trial. Our study shows that regular water supply is an important aspect of vector ecology and mosquito control, with or without community mobilisation. The numbers of water storage containers kept by the households reflected within-neighbourhood variations in water supply.

Solving problems of water supply requires medium- and long-term investments. Meanwhile, households obliged to store water should prevent mosquitoes from using their storage containers to reproduce. The community mobilisation intervention of Camino Verde reduced *Aedes aegypti* indices in households with regular water supply as well as those with irregular water supply.

The informed community mobilization, aimed at reducing mosquito breeding sites in and around households, made a significant difference to households that reported regular water supply, but it did not eliminate entomological risk. A possible explanation is that “regular” supply was not adequate for all household needs. We found water storage barrels in 9% of households that reported “regular” water supply. These households may not have had full confidence in their water supply and therefore kept water barrels for emergencies. Furthermore, households with a regular water supply still have a risk of dengue from other dynamics. They live close to neighbouring households and may be in range of the adult mosquitoes propagated there. *Aedes aegypti* is also active in daylight hours; people can be exposed as they move out of immediate home and neighbourhood environments where control is adequate [12].

### Limitations

Entomology inspection teams did not participate in the trial intervention, but we cannot exclude the possibility they became aware of which households were in intervention and which in control clusters. Since we used household as the unit of analysis, to match the household water supply, we could not analyse the relationship with containers (Breteau and Container Index) or population density (pupa per person). The two indicators we could use in a household analysis reflected either all immature forms (House Index) and or pupae specifically (Pupae-positive households). Dichotomising variables can introduce problems of interpretation, especially if a negative result could be an effect of an arbitrary dichotomy. Our interpretation hinges on the fact the same dichotomy was used for all subgroups. Our sensitivity analysis with additional cut-off points for water supply regularity obtained substantively the same results as the main analysis.

## Conclusion

While *Aedes aegypti* control efforts based on informed community mobilisation might prioritise communities with difficulty accessing a regular water supply, the intervention also impacts entomological indices of households with a regular water supply. These households should not be excluded from community mobilisation efforts to reduce the *Aedes aegypti* vector.

## Abbreviations

CNDR: *Centro Nacional de Diagnóstico y Referencia* (National Diagnostics and Reference Centre at the Ministry of Health)
ENACAL: *Empresa Nacional de Acueductos y Alcantarillados* (National Water and Sewage Company)
SILAIS: *Sistema Local de Atención Integral en Salud* (Public health service for the Department of Managua)
